# Obituary

**Published:** 1889-06

**Authors:** 


### Obituary.

Dr. C. B. Porter was born at Rochester, New York, on the 24th of April, 1820, and died at Bay City, Michigan, March 20th, 1889, being nearly sixty-nine years old at the time of his death.

In 1832 he came to Michigan, then a territory, and with his father cleared up a homestead in Sharon township, Washtenaw county, where he lived until 1850 when he married Abigail Sawyer, at Jackson, Michigan, who survives him.

In the fall of 1850 he entered the Medical Department of the University of Michigan, and attended one course of lectures, his idea at that time being to make diseases of the eye a specialty. Soon after, however, feeling that dentistry opened up a larger field he adopted that profession as his life vocation. He took such instruction as was available at that time including a course under Dr. John Allen in Continuous Gum-Work, and opened offices in Ann Arbor, Saline, and Grass Lake, making each place at intervals of a few weeks.

The probability that Ann Arbor would become the most important place in the county induced him to give up his Saline and Grass Lake offices and devote his whole attention to his rapidly increasing Ann Arbor practice.

It was about this time in his life that he with Dr. Metcalf, of Kalamazoo, and Dr. Whiting, of E. Saginaw, commenced to agitate the advisability of a State Dental Association, from which the present flourishing society is the outgrowth. Some of the early meetings were held at his residence in Ann Arbor.

After twenty-nine years practice at Ann Arbor he located at Bay City and for ten years had been in active practice there

Although he had nearly reached the full time allotted for man, yet he never lost interest in his chosen work and was ambitious to introduce into his operating room and laboratory every new device of merit whereby he could better serve those who called on him. Dr. Porter was a man of a noble nature, congenial, and esteemed and loved by all who knew him. He was a model Christian man whose loving heart and kindly sympathy was ever extended to every worthy object. The writer knew Dr. Porter personally for twenty-five years and never found in him any thing but true noble manhood, in the highest sense.

